# Joint Rhythmic Movement Increases 4-Year-Old Children’s Prosocial Sharing and Fairness Toward Peers

**DOI:** 10.3389/fpsyg.2017.01050

**Published:** 2017-06-26

**Authors:** Tal-Chen Rabinowitch, Andrew N. Meltzoff

**Affiliations:** Institute for Learning & Brain Sciences, University of Washington, SeattleWA, United States

**Keywords:** synchrony, music, sharing, children, rhythmic movement, prosocial behavior, fairness, social cognition

## Abstract

The allocation of resources to a peer partner is a prosocial act that is of fundamental importance. Joint rhythmic movement, such as occurs during musical interaction, can induce positive social experiences, which may play a role in developing and enhancing young children’s prosocial skills. Here, we investigated whether joint rhythmic movement, free of musical context, increases 4-year-olds’ sharing and sense of fairness in a resource allocation task involving peers. We developed a precise procedure for administering joint synchronous experience, joint asynchronous experience, and a baseline control involving no treatment. Then we tested how participants allocated resources between self and peer. We found an increase in the generous allocation of resources to peers following both synchronous and asynchronous movement compared to no treatment. At a more theoretical level, this result is considered in relation to previous work testing other aspects of child prosociality, for example, peer cooperation, which can be distinguished from judgments of fairness in resource allocation tasks. We draw a conceptual distinction between two types of prosocial behavior: resource allocation (an other-directed individual behavior) and cooperation (a goal-directed collaborative endeavor). Our results highlight how rhythmic interactions, which are prominent in joint musical engagements and synchronized activity, influence prosocial behavior between preschool peers.

## Introduction

Music is an essential ingredient of human life in all human cultures. It has long been recognized that music influences people’s psychology in profound ways, as discussed by Plato (2006), Aristotle (1941), and Schopenhauer (1966). In the last few decades, experimental research has revealed the causal effects of music on cognitive, social, and emotional functioning (e.g., Hallam et al., 2011; Fancourt et al., 2014; Clarke et al., 2015). Perhaps one of the most primal functions of music is social bonding. Whether it is an ancient ritual, a stadium chant, or a sing-along, music enables friends or even strangers to close the distance between self and other.

### Music and Human Society

How does music facilitate social accord between people? Several features of music enhance its social functions. First, music is a flexible medium for non-verbal communication. It facilitates creative exchange between individuals that does not depend on language. Creating music with partners is abstract, interpretive, and concurrent (participants engage simultaneously)—promoting a feeling of sharing and communality (Cross, 2001). Second, music readily evokes body movements, such as tapping, nodding, and dancing. Moreover, producing music, whether through singing or the use of physical instruments, typically involves overt body motions to generate the sound. Third, music is fundamentally rhythmic, containing repeated patterns of sound and therefore involves recurring patterns of rhythmic movements. Taken together, such repetitive joint rhythmic action may be especially effective in tapping interpersonal similitude and coordination (e.g., Dalla Bella et al., 2013), which could potentially foster intersubjectivity (Feldman, 2007; Gallagher, 2008; Gallagher and Hutto, 2008), in part because it contains basic imitative elements (Meltzoff, 2007, 2013; Saby et al., 2012).

Importantly, rhythmic movements during music can be synchronous (temporally aligned), semi-synchronous, or at times even asynchronous (temporally incongruous). Synchrony enables participants to coordinate their motions and move as one. Asynchrony, such as in polyphonic (e.g., Huron, 2008) and polyrhythmic (e.g., Poudrier and Repp, 2013) music, introduces an added layer of complexity, emphasizing to individual participants that they are taking part in a larger synergistic joint composition.

### Music, Synchrony, and Prosociality in Children

Studies suggest that music can positively affect social behaviors of children. For example: (i) kindergartners are more likely to choose to cooperate with another partner than to play by themselves following a shared musical experience, as opposed to a shared non-musical experience (Kirschner and Tomasello, 2010), (ii) repeated sessions of joint music-making enhance elementary school children’s emotional empathy compared to verbal play controls (Rabinowitch et al., 2013), (iii) elementary school children’s singing is associated with a self-reported sense of social inclusion (Welch et al., 2014), and (iv) children undergoing musical training in elementary school tend to be more sympathetic to others, according to self-report questionnaires (Schellenberg et al., 2015).

Researchers have also explored the specific impact of rhythmic interaction (independent of a musical setting) on the social behavior of infants and children by comparing synchronous to asynchronous interactions. These studies suggest that synchrony as opposed to asynchrony selectively increases collaborative cooperation (Rabinowitch and Meltzoff, 2017), helping behavior (Cirelli et al., 2014; Tunçgenç and Cohen, 2016a), prosocial attitudes (Rabinowitch and Knafo-Noam, 2015), and social bonding (Tarr et al., 2015; Tunçgenç and Cohen, 2016b). Related effects of synchrony have also been reported in adults (e.g., Macrae et al., 2008; Hove and Risen, 2009; Miles et al., 2009; Lakens and Stel, 2011; Valdesolo and DeSteno, 2011).

### The Special Case of Sharing Behavior

Sharing behavior, and in particular, other-directed resource allocation is an important form of prosocial behavior, which can be distinguished on theoretical and empirical grounds from other types of prosocial behavior. The sharing of resources and sense of fairness that often drives it changes with age (Fehr et al., 2008). Sharing behavior is itself malleable and influenced by prior experience and context, as shown in previous studies manipulating reciprocal activity such as rolling a ball together or pushing buttons to activate a toy (Barragan and Dweck, 2014), pulling a rope together (Hamann et al., 2011; Warneken et al., 2011), or performing repeated iterations of the Prisoner’s Dilemma (Blake et al., 2015; Sebastián-Enesco and Warneken, 2015). Can children’s sharing behavior be affected by music or other shared rhythmic behavior?

The effects of music on children’s sharing behavior are not clear. Good and Russo (2016) reported that group singing among elementary school children increased their propensity to share with each other in a Prisoner’s Dilemma game. However, when children aged two to four performed joint drumming with an adult, no changes in sharing behavior toward the adult experimenter were found (Kirschner and Ilari, 2014).

It remains untested whether rhythmic interaction, in the absence of music, can change how children allocate resources between themselves and a peer—inducing sharing and generosity. This is an important question because considering others when distributing resources is a key aspect of human social interaction and culture, related to altruism, fairness, and ultimately to human ethics (Fehr et al., 2008).

### Rationale for the Current Study

The current study aimed to explore how joint rhythmic movement of young children affects their subsequent sharing behavior toward an unfamiliar peer. In Fehr et al. (2008), sharing behavior was tested both between children who were well acquainted and those who had never met before, and there was a difference. In the current work, we examined same-sex pairs of 4-year-olds who were strangers prior to the study.

In order to measure sharing, we employed a paradigm designed to probe resource allocation and the sense of fairness among children (Fehr et al., 2008). This was done by presenting participants with a sequence of two-forced choice decisions about resource allocation. We adopted the original choice patterns developed by Fehr and colleagues and used them to distinguish between variants of children’s sharing behavior. We tested whether a participant made generous sharing choices and at what expense to the self. Were participants willing to give away part of their own allocation and distribute resources unequally between themselves and peer? The use of Fehr’s procedure and outcome measures enabled us to test the impact of our designed treatment involving rhythmic interaction on precise aspects of resource allocation and sharing behavior in preschool children.

To administer the rhythmic intervention, we developed a uniquely precise swinging apparatus (Rabinowitch and Meltzoff, 2017). Using this device, we administered rhythmic swinging experience to pairs of previously unacquainted 4-year-olds. This treatment was either synchronous or asynchronous, and we then compared sharing behavior as a function of these two conditions and an additional baseline condition of no swinging.

Children in the current study also performed collaborative problem-solving tasks involving cooperation, as reported in Rabinowitch and Meltzoff (2017). In that study, we found that synchronous but not asynchronous rhythmic movement resulted in enhanced performance of the cooperative tasks. In the present paper, we examine a new and different dependent measure, one involving other-directed resource allocation, as tested by Fehr et al.’s (2008) tasks.

Sharing behavior and cooperation are both prosocial behaviors and may be related (Hay, 1979; Tomasello et al., 2005; Fehr et al., 2008; Olson and Spelke, 2008; Warneken et al., 2011). Therefore, one possible prediction for the current study would be that since experience with synchrony enhanced 4-year-olds’ goal-directed cooperation more than experience with asynchrony did (Rabinowitch and Meltzoff, 2017), synchrony would in the same way enhance their sharing.

However, there are also several conceptual differences between other-directed sharing and goal-directed cooperation that might lead to a difference in how they are influenced by experiences of synchrony and asynchrony. In particular, cooperation often requires joint action and temporal coordination, and is directed toward a common goal of successful task completion. On the other hand, sharing is accomplished by an individual, does not rely on temporal coordination, and is not motivated by achieving a concrete joint goal. Therefore, an alternative prediction could be made that the experience of joint rhythmic synchrony and asynchrony could equally enhance sharing behavior. That is, the specific features of synchrony might be less crucial for affecting sharing than for influencing cooperation.

As noted earlier, joint musical activity consists of rhythmic movement that may be synchronous, partially synchronous, and at times even asynchronous. The apparatus that we designed to swing pairs of 4-year-old peers made it possible to study both synchronous and asynchronous rhythmic movement outside of a musical context. In the current study, the rhythmic movement experienced by the children did not stem from an active intention to move with their peer, as occurs during joint music making. Rather it was randomly assigned and controlled by the experimenters. This enabled us to examine the effects of rhythmic interaction—synchronous and asynchronous movement in contrast to a baseline control—on 4-year-old children’s resource allocation.

In sum, this experiment testing the effects of specially designed treatments on sharing and the allocation of desirable resources serves to: (i) extend our knowledge about prosocial behavior among preschool children and (ii) inform us about the malleability of sharing as a function of a short-term laboratory intervention.

## Materials and Methods

This study was carried out in accordance with the recommendations of the University of Washington’s Human Subjects Division, and was approved by the University of Washington’s Institutional Review Board. All parents of children gave written informed consent for their children to participate in this study and all children gave oral informed assent in accordance with the Declaration of Helsinki.

### Participants

The cohort of children in this study was the same as that in Rabinowitch and Meltzoff (2017), but the current paper reports different dependent measures. The overall sample consisted of *N* = 162 typically developing 4-year-old children (*M*_age_ = 53.21 months, *SD* = 3.06) paired into same-sex dyads. None of the children had met each other before the test. Additional dyads were excluded due to unwillingness to use our apparatus (*n* = 2) or tiredness/unwillingness to continue of one or both dyad members (*n* = 4). Pre-established criteria for admission into the study were that the children were typically developing and had no known developmental concerns. According to parental report, the sample was middle- to upper middle-class, with 71% White, 4.9% Asian, 0.6% African-American, 20.4% mixed race, 3.1% not disclosed; 11.7% of the participants were of Hispanic ethnicity.

### Design

Children were randomly assigned in equal numbers to one of three independent groups: Synchrony, Asynchrony, or Baseline. Each group was composed of 27 dyads (14 of which were female dyads). Sample size was chosen based on a related study with a similar number of dyads (Rabinowitch and Knafo-Noam, 2015). All children participated in randomly ordered rounds of testing, each consisting of a joint rhythmic interaction (except for the Baseline group), which was followed by a behavioral resource allocation task.

### Apparatus

**FIGURE 1:**
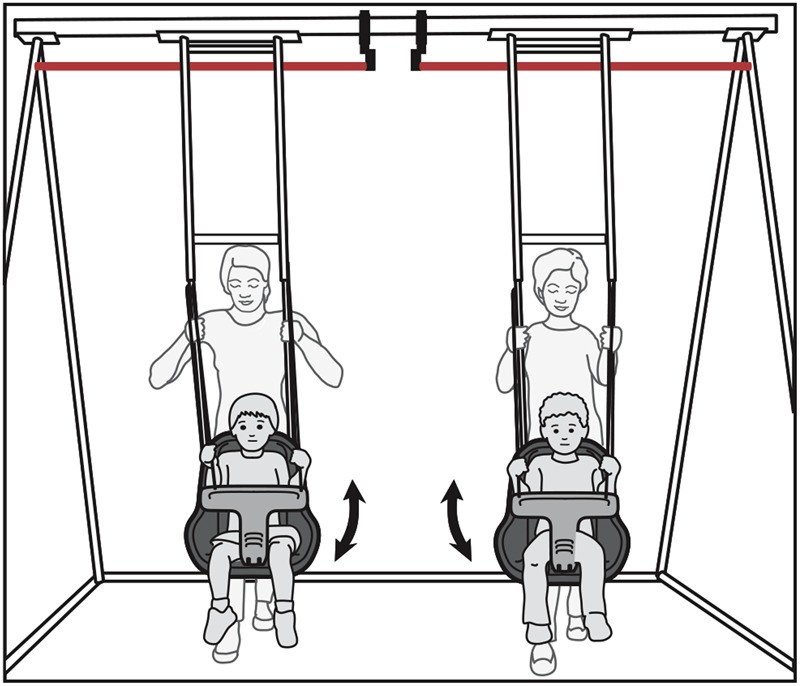
Swing-set apparatus. Illustration of two 4-year-old peers swinging together. An infrared beam (red line) fed time-stamps to a computer each time the beam was broken by the swing (from Rabinowitch and Meltzoff, 2017).

We constructed a swing-like apparatus that could move two children together in space (**Figure 1**). The movement of the swings was electronically measured to confirm the precision of the intervention. The swing-set was operated by two trained experimenters who pushed the swings according to the timing of two bouncing balls with beeps indicating when the swings were supposed to cross the 0 point. In the synchronous group, the child peers were swung in unison (i.e., at the same rate and in phase with each other, at a cycle time of either 2.0 or 2.6 s determined by random assignment). In the asynchronous group the child peers were swung at different paces (i.e., one child in the dyad was swung at a cycle time of 2.0 s and the other at a 2.6 s cycle time, determined by random assignment). Children in the baseline group were not swung at all.

### Procedure

Pairs of same-sex children who had never before met were randomly assigned to one of three experimental groups. (a) The *Synchronous group* experienced synchronous joint rhythmic movement. (b) The *Asynchronous group* experienced asynchronous joint rhythmic movement. (c) The *Baseline group* was not administered any rhythmic experience and served as a control for measuring children’s performance in the absence of any treatment. Following the treatment each dyad was administered the Sharing tasks (as well as two goal-directed cooperative tasks reported in Rabinowitch and Meltzoff, 2017).

For the Synchrony and Asynchrony groups, each task was administered in three phases: (a) demonstration of the behavioral task, (b) swinging treatment (2.5 min), and (c) a test period assessing children’s performance on the behavioral task. Children from the baseline group were administered phase-a and then directly phase-c. The Sharing tasks were demonstrated and explained to the children prior to treatment in order to minimize the time between the treatment (e.g., synchrony experience) and the test period on the task. Test sessions were video-recorded.

The two cooperative tasks from Rabinowitch and Meltzoff (2017) required simultaneous coordination between the peers to achieve a joint goal. These two tasks and the sharing tasks to be reported here were presented in a counterbalanced order across children. (We also tested whether the results varied as a function of the order of the tasks, and found no such order effect, see Results.)

#### Demonstration and Test Periods for the Resource Allocation Test

The resource allocation test, which we adapted from Fehr et al. (2008), was used to examine the effects of rhythmic movement on sharing behavior. We separated the two child participants for the duration of this assessment, so that they could not see or hear each other’s choices while being tested. In this way, we ensured that the children’s decision-making process would be unbiased by their partner’s reactions.

The resource allocation test consisted of a series of three consecutive tasks, concerning the distribution of attractive toy bears (2.5 cm tall) between self and partner. In each task, children made a two-alternative forced-choice response for how to distribute the toys between self and other. These three tasks were originally named in Fehr et al. (2008) as “Prosocial,” “Envy,” and “Sharing.” Here, we renamed them simply as “Sharing I,” “Sharing II,” and “Sharing III.” (We chose to use attractive toy bears instead of the sweets used by Fehr et al. to avoid hesitancy or refusal to participate in the study by health-conscious parents.)

**FIGURE 2:**
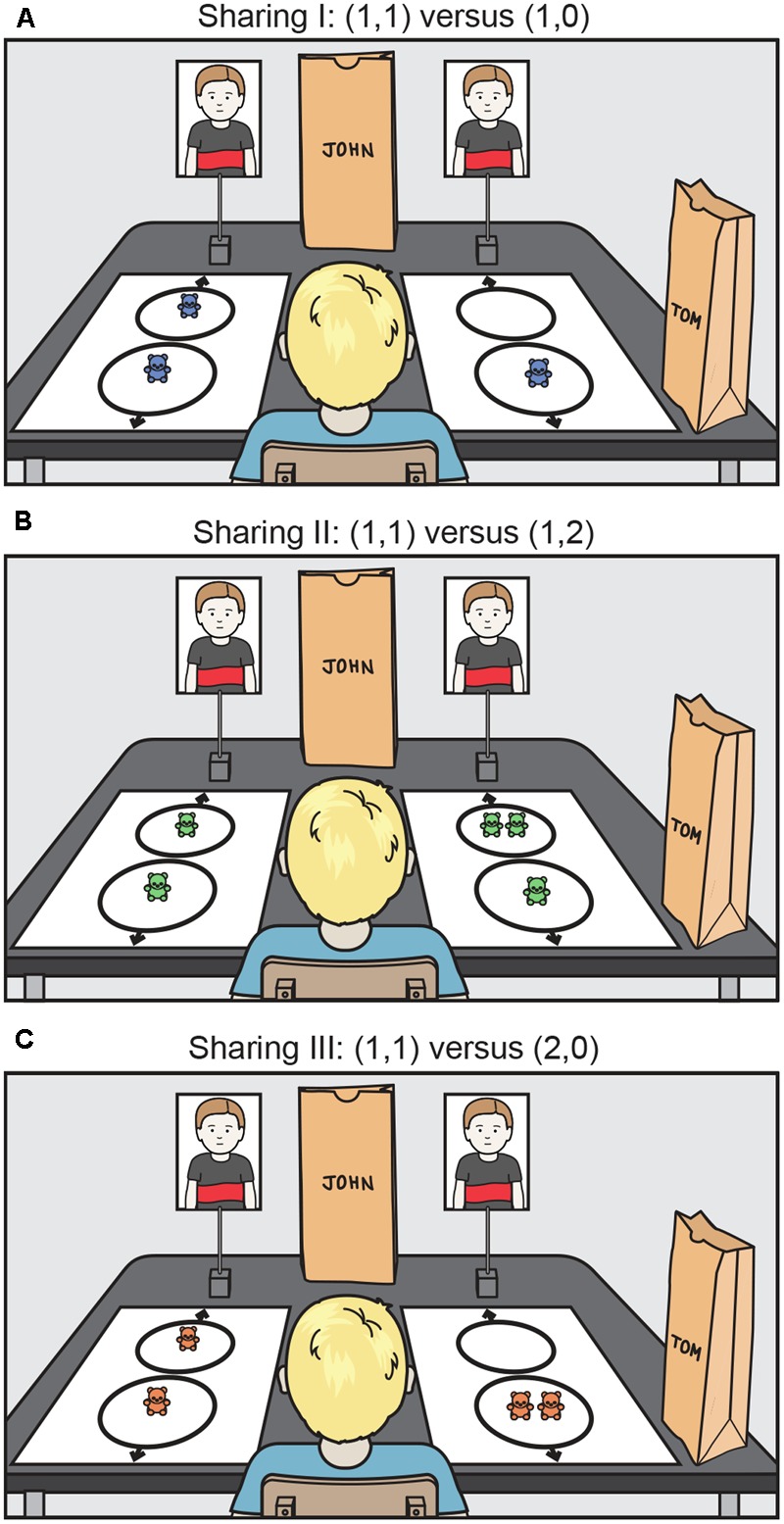
The test used to measure child sharing. The participant is in the foreground, and two photographs of the peer are at the far end. In this example, the participant is Tom, and the peer is John. The participant’s task was to choose one of the distributions (shown in the white rectangles; left/right positioning counterbalanced). The near circle contained the bears for the self; the far circle contained the bears for the peer. Thus, in **(A)** (left rectangle) there was one bear for the self and one for the other, as represented by (1,1). The choices were as follows: **(A)** Sharing I: (1,1) versus (1,0), **(B)** Sharing II: (1,1) versus (1,2), and **(C)** Sharing III: (1,1) versus (2,0). After the participant made a choice, the appropriate number of bears were deposited in the bags (and the bears for the unselected choice were removed from the table). The other two Sharing tasks were administered in the same manner. These tasks were adapted from Fehr et al. (2008).

As shown in **Figure 2**, one of the options for each task presented to the child was more generous than the other (i.e., it increased the peer’s share). In Sharing tasks I and II, the generous choice did not affect the self’s share of resources, but provided more resources to the peer than the non-generous one. In Sharing task III, a generous choice required the child to reduce his or her own share and increase the share to the peer.

The two alternatives in each task were presented side by side on a table. The relevant resources (toy bears) to be allocated to each individual (participant and peer) were placed between the participant (self) and peer (other), as indicated in **Figure 2**. Thus each participant faced an array that showed two possible distributions of resources, and they were asked to select one of them: “Which would you like to choose?” Each participant received two brown paper bags, one with their own name written on it and the other with the peer’s name. The participant was told that during the task he/she would decide how the bears would be distributed. The child indicated his/her choice by pointing. The child was informed that the resources (bears) chosen would then be placed in the respective brown bags and would be taken home by the participant and the (now absent) peer. Which bag was for the self and which for the other was clearly marked with the children’s name written in hand on the bag (**Figure 2**).

During the practice demonstration, the experimenter explained the rules of the game, followed by a series of questions intended to confirm that the child indeed understood them. As a practice task, the child was asked to select between two sets of equal numbers of bears such that the arrays differed only in color (e.g., red versus blue). The bears were then placed in the corresponding bags for self and other. During the formal testing, each participant was presented with a sequence of the three sharing tasks (order counterbalanced across children) involving bears of the same color (the test involved colors that were different from the practice colors; altogether, there were six colors, counterbalanced across the tasks and children tested). We used bears of different colors to introduce, as did Fehr et al. (2008), some surface-level variation in the tasks (Fehr et al. used different kinds of sweets). After completing all the experimental tasks, children were asked whether they were happy with the number of bears that they had obtained and if they thought their partner would be happy with the number of bears that he or she received.

### Dependent Measures

We used two measures. The first was the percentage of individual children making the generous sharing choice in each task (the generous sharing choice was always the one that maximized the number of bears for the peer, see **Figure 2**). We also derived a dyad sharing score. If neither of the participants in the dyad chose generously, the dyad’s score was 0. If only one member presented a generous response, the score was 1. If both members of a dyad showed a generous response, the dyad received a score of 2.

Videotapes of children’s test performance were scored by two coders. Scoring agreement was measured for a random sample of 25% of the children. There were no disagreements for either intra- or inter-scorer agreement on either measure (Cohen’s *kappa* = 1.00). A small portion of tasks (<1%) could not be scored.

## Results

**FIGURE 3:**
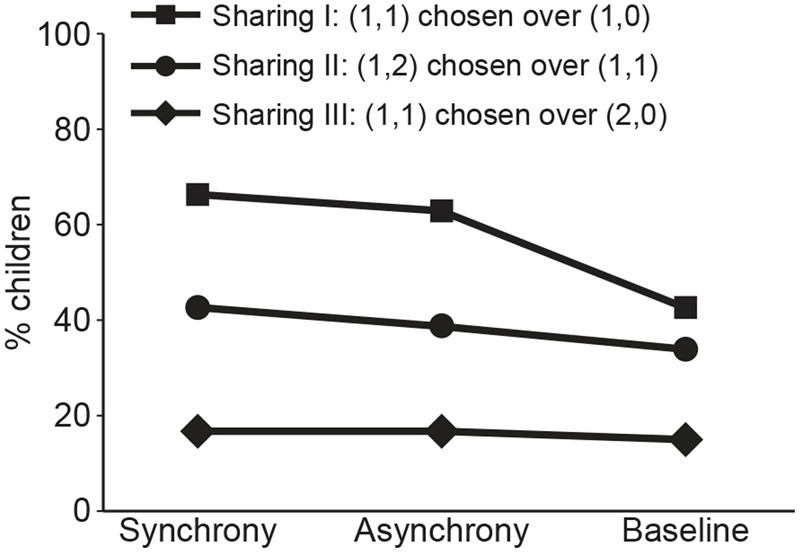
Percentage of children exhibiting the generous sharing choice for each of three sharing tasks as a function of experimental group (Synchrony, Asynchrony, and Baseline).

First we examined the behavior of the children who were randomly assigned to the baseline group (**Figure 3**). This was done to compare our current results to those reported in Fehr et al. (2008) who also used peers who had not met before, similar to our baseline group (which Fehr et al. termed a test of “outgroup” members). In agreement with that study, children in our baseline group exhibited more generous sharing choices in the Sharing I (43%) and Sharing II (34%) tasks than in the Sharing III task (15%). This result in our no-treatment group, which is consistent with Fehr et al.’s findings, provides a baseline level against which to compare the effects of our treatment of joint rhythmic experience.

We next examined how our treatment influenced the behavior on the Sharing I task (**Figure 3**). A logistic regression including experimental condition and gender as factors showed that the number of children who made generous choices varied significantly as a function of experimental condition, χ^2^ (3, *N* = 161) = 7.05, *p* = 0.03. *Post hoc* comparisons revealed a significant difference between the synchrony group (67% of participants chose the generous allocation) and the baseline group (43% did so), *B* = 0.99; *p* = 0.01, as well as between the asynchrony group (63% of participants chose the generous allocation) and the baseline group, *B* = 0.82; *p* = 0.04. There was no significant difference between the synchrony and asynchrony groups, *B* = -0.16; *p* = 0.68. There was no significant main effect for gender (*p* = 0.07) and no significant condition by gender interaction (*p* = 0.91).

The Sharing II task, in which a generous choice required an unequal distribution between self and peer, showed no significant difference between the synchrony, asynchrony, and baseline groups [43, 39, and 34%, respectively, logistic regression χ^2^ (3, *N* = 161) = 0.84, *p* = 0.66]. Similarly, the Sharing III task, in which a generous choice entailed a smaller share for self, yielded consistently low levels of generous sharing and no difference between groups [17, 17 and 15%, respectively, logistic regression χ^2^ (3, *N* = 161) = 0.07, *p* = 0.96]. There were no significant main effects of gender (*p*s > 0.14) or gender by condition interactions (*p*s > 0.31) on these tasks.

**FIGURE 4:**
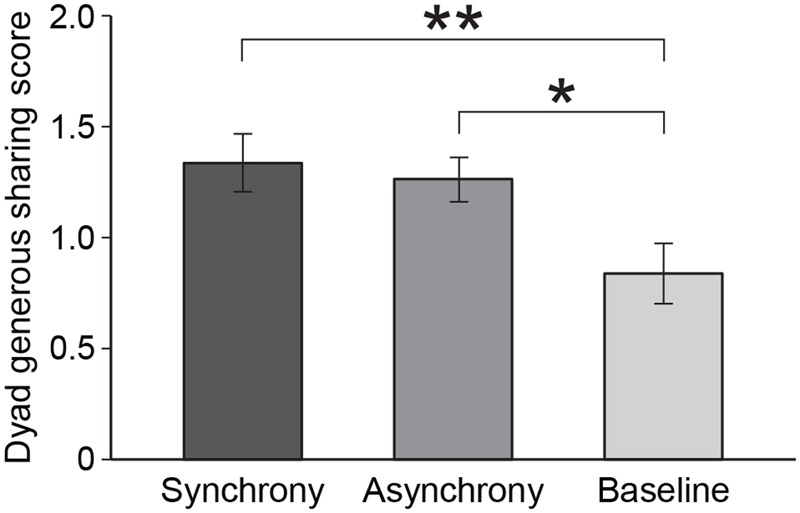
Dyad generous sharing score. Each dyad was given a score of 0, 1, or 2 in accordance with whether neither dyad member, one dyad member, or both dyad members displayed a generous sharing choice in the Sharing I task. Recall that the dyad members were tested separately and therefore did not know whether the peer did, or did not, choose to share the resources. ^∗^*p* < 0.05, ^∗∗^*p* < 0.01.

Because we observed an effect of rhythmic interaction on children’s generous sharing choices in the Sharing I task, we sought to examine whether such an effect was also apparent at the dyad level. We assigned a score of 0, 1, or 2 to the dyad according to how many members of the dyad made the generous choice in this task (see Materials and Methods). A two-way analysis of variance with experimental condition and gender as factors showed a significant main effect of experimental condition on these dyad-level sharing scores, *F*(2, 74) = 4.80, *p* = 0.01 (a non-parametric Kruskal–Wallis test yielded a similar result, χ^2^ (2, *N* = 80) = 8.1, *p* = 0.02). *Post hoc* pairwise comparisons using Fisher’s least significant difference (LSD) procedure showed no difference in dyad sharing scores between children swung synchronously (*M* = 1.3) and asynchronously (*M* = 1.2), *p* = 0.66, and each of these scores were significantly greater than the baseline score (*M* = 0.8), *p* = 0.006 and *p* = 0.02, respectively, **Figure 4**. There was also a main effect of gender, *F*(1,74) = 4.44, *p* = 0.04, with girls showing more sharing than boys, and no condition by gender interaction (*p* = 0.90). In addition, we conducted a related test to address this issue by comparing the number of dyads in which *both* members made generous choices (a score of “2” indicating a high-sharing dyad) versus dyads in which *neither* member chose generously (a score of “0” indicating a low-sharing dyad). As expected, a logistic regression analysis showed that high versus low-sharing varied significantly as a function of experimental condition, χ^2^ (3, *N* = 36) = 8.87, *p* = 0.01, with more high-sharing dyads in both the synchrony and asynchrony treatment groups than in the baseline group (*p*s = 0.03) and no difference between the two treatment groups (*p* = 0.63). This main effect of experimental condition on sharing is of special interest because the children were tested separately on this task and had no access to each other’s choices. There was also a main effect of gender (*p* = 0.04) and no condition by gender interaction (*p* = 0.65) for this measure. (Because the gender effect did not emerge for some measures in this paper and there was no gender by experimental condition interaction on any measure, we do not interpret it further at this time.)

We also wondered whether children increased their generosity in the Sharing I task purely as a function of the degree of “familiarity” with each other, and therefore we analyzed children’s generous sharing as a function of when it occurred in the session. In other words, children were strangers to begin with but might gradually become more familiar with each other as they swing and perform more tasks over time. A 3 (test position: 1st/2nd/3rd task) by 2 (test condition: baseline/treatment) statistical test was used for both measures reported earlier, that is, the percentage of children showing generous sharing and the dyad sharing score. For both, there was a significant main effect for test condition as expected (*p*s < 0.01), no main effect for test order (*p*s > 0.21), and no interaction between test order and test condition (*p*s > 0.70). This suggests that aspects of the treatment condition (e.g., rhythmic movement) rather than solely increased familiarity with the peer over the course of the test was the key factor.

Finally, after completing the resource allocation tasks children were asked whether they “were happy” with the number of bears they obtained. Overall, 99% of participants responded that they were. When asked whether the *other* child would be happy with the number of bears he or she would be receiving, 79% of participants who made a generous choice in the Sharing I task responded positively, 14% responded negatively, and 7% were undecided. For those who made a non-generous sharing choice, 48% responded positively, 43% negatively, and 9% were undecided, χ^2^ (2, *N* = 161) = 17.81, *p* = 0.0001. Thus, there seemed to be some awareness by the children of the impact of their choices on their peers. This is interesting because the peers were not in the room at the time, and yet the children’s representation of the absent peer included an emotional attribution. Further manipulations are needed to pursue these effects in more detail, but the current findings fit together with results in the literature about children’s psychological attributions concerning gift-giving (e.g., Atance et al., 2010).

## Discussion

Our results show that a brief encounter between previously unacquainted 4-year-old children, consisting of either synchronous or asynchronous rhythmic movement, was sufficient to alter their sharing behavior compared to children who did not undergo any treatment. Previous research has shown that children more readily share resources following a short reciprocal interaction (Barragan and Dweck, 2014), or after a collaborative effort which possibly elevates their sense of social, collaborative justice (Hamann et al., 2011). Our results showing an increase in sharing choices following a joint, rhythmic swinging experience are consistent with these findings.

Notably, children who experienced joint rhythmic movement (either synchronous or asynchronous) increased their tendency to allocate resources to their swinging partner in a generous fashion (Sharing I task), as long as this did not require them to distribute the resources unequally (Sharing II) or to reduce their own share (Sharing III task).

Interestingly, Fehr et al. (2008) reported a related result. They found that 4-year-olds performing the Sharing I task showed more frequent generous sharing choices toward “ingroup” peers. Conceptually, this fits with our finding of more generous sharing choices toward peers after joint rhythmic movement compared to no treatment (**Figure 3**, Sharing I task). In contrast, when challenged with the need to unevenly allocate resources (Sharing II task) or to reduce one’s own share of resources by “giving something up” to the peer (Sharing III task), neither the ingroup (Fehr et al., 2008) nor a shared rhythmic treatment (**Figure 3** in current study) was sufficient to induce generous sharing under these conditions. In sum, by 4 years of age, children’s likelihood of generously allocating resources is malleable (e.g., modulated by a brief preceding session of joint rhythmic movement), but only if the cost of this sharing behavior does not result in an unequal distribution of resources or giving away one’s own share of resources.

Could the observed change in generous sharing be due solely to the time that the children spent together during the test rather than to the joint rhythmic movement? Our analysis showing no effect of task position argues against this possibility. If increased acquaintance was the sole driver of our result behavior, then children presented with the sharing task in the first position would have shown less generous choices, but that did not occur. Nevertheless, in future work it would be informative to add a control condition whereby children experience joint play and familiarity in a way that does not involve rhythmic movement, to assess the degree to which the obtained effect is due to the rhythmic experience *per se*.

We now wish to go beyond the current data and to consider it in relation to previously published work in order to offer speculations about differentiations in kinds of prosocial activity. Recall that children in the current study were also administered two cooperation tasks (see Materials and Methods). As we previously reported, the children showed greater cooperation following the synchronous treatment versus the asynchronous treatment or the baseline no-treatment control (Rabinowitch and Meltzoff, 2017). This indicates that the children’s cooperative behavior (unlike the current sharing behavior) significantly varied as a function of the particular temporal structure of the swinging (synchronous versus asynchronous). We are reporting our results in two papers because the tasks and dependent measures are very different, and in the remainder of the discussion we provide theoretical speculations about potentially important distinctions between sharing and cooperation.

Cooperating with a peer (Rabinowitch and Meltzoff, 2017) and sharing of resources with a peer (the current report) are both prosocial behaviors broadly construed (Hay, 1979; Tomasello et al., 2005; Fehr et al., 2008; Olson and Spelke, 2008; Warneken et al., 2011). It is therefore of interest that for the *same* participants, cooperation was enhanced specifically by synchrony, whereas sharing was enhanced by both synchrony and asynchrony, that is by both rhythmic activities. These results suggest a possible dissociation between sharing versus collaborative cooperation.

Several differences between the two might explain this pattern of results. First, the performance of a cooperative task depended on *dyad* behavior rather than individual behavior, and may thus be more influenced by paired synchronized movement of the dyad as a unit. In order to cooperate, the members have to be sensitive to the other person’s actions and adjust their behavior accordingly in real-time. In contrast, resource allocation and generosity have a strong individual aspect, especially when evaluated with the sharing tasks used in the current study. Crucially, the allocation of resources occurred in the absence of the peer partner and was an individual and private choice.

Second, most cooperation inherently involves some degree of temporal coordination or congruence which is also embodied in the synchronous movement treatment. For example, in Rabinowitch and Meltzoff (2017) participants were administered cooperation tasks that required both dyad members to execute either simultaneous actions (pressing two buttons at the same time) or complementary actions (one participant passing an object and the other receiving it). In contrast, individual decision-making about resource allocation and fairness does not depend on such congruent action or alignment.

Third, sharing behavior *per se* (especially as manifested by the current resource allocation tasks) could be driven by altruistic or egalitarian motives, whereas the cooperative tasks had a concrete goal in the physical world—through cooperating with another, the children achieve an outcome or goal they could not achieve by themselves. Speculatively, it is possible that shared rhythmic movement alters resource allocation behavior because it induces a change in attitude about the peer (e.g., perhaps the treatment creates a sense of ingroup positivity due to the shared experience). This may not be sufficient, however, to enhance cooperation. Instead, working cooperatively to achieve a goal through coordinated action might be particularly enhanced by joint *synchronous* experience—an activity in which the dyad moves “as one” in congruence and alignment with one another. This suggests that the effects of rhythm and synchrony (and perhaps music) on the subsequent behavior of young children are not uniform across different types of prosocial responses and may involve differentiable social-cognitive mechanisms. These can be dissected through future experimentation, with implications for theories of social cognition and the design of childhood interventions.

## Author Contributions

T-CR and AM designed the study. T-CR tested the children. T-CR and AM analyzed the data and wrote the manuscript.

## Conflict of Interest Statement

The authors declare that the research was conducted in the absence of any commercial or financial relationships that could be construed as a potential conflict of interest.
